# Metabolic Profiling of Pyrrolizidine Alkaloids in Foliage of Two *Echium* spp. Invaders in Australia—A Case of Novel Weapons?

**DOI:** 10.3390/ijms161125979

**Published:** 2015-11-06

**Authors:** Dominik Skoneczny, Paul A. Weston, Xiaocheng Zhu, Geoff M. Gurr, Ragan M. Callaway, Leslie A. Weston

**Affiliations:** 1 Graham Centre for Agricultural Innovation, Charles Sturt University , Wagga Wagga, NSW 2678, Australia; dskoneczny@csu.edu.au (D.S.); pweston@csu.edu.au (P.A.W.); xzhu@csu.edu.au (X.Z.); ggurr@csu.edu.au (G.M.G.); 2 Institute of Applied Ecology, Fujian Agriculture & Forestry University, Fuzhou 350002, China; 3 Division of Biological Science, University of Montana, Missoula, MT 59812, USA; ray.callaway@mso.umt.edu

**Keywords:** metabolomics, UHPLC Q-TOF, Paterson’s curse, *E. plantagineum*, *E. vulgare*, plant defense

## Abstract

Metabolic profiling allows for simultaneous and rapid annotation of biochemically similar organismal metabolites. An effective platform for profiling of toxic pyrrolizidine alkaloids (PAs) and their *N*-oxides (PANOs) was developed using ultra high pressure liquid chromatography quadrupole time-of-flight (UHPLC-QTOF) mass spectrometry. Field-collected populations of invasive Australian weeds, *Echium plantagineum* and *E. vulgare* were raised under controlled glasshouse conditions and surveyed for the presence of related PAs and PANOs in leaf tissues at various growth stages. *Echium plantagineum* possessed numerous related and abundant PANOs (>17) by seven days following seed germination, and these were also observed in rosette and flowering growth stages. In contrast, the less invasive *E. vulgare* accumulated significantly lower levels of most PANOs under identical glasshouse conditions. Several previously unreported PAs were also found at trace levels. Field-grown populations of both species were also evaluated for PA production and highly toxic echimidine *N*-oxide was amongst the most abundant PANOs in foliage of both species. PAs in field and glasshouse plants were more abundant in the more widely invasive species, *E. plantagineum*, and may provide competitive advantage by increasing the plant’s capacity to deter natural enemies in its invaded range through production of novel weapons.

## 1. Introduction

Metabolites are the end products of gene expression and are a reflection of an organism’s state at a particular moment in time [1,2,3]. Metabolites directly reflect the interaction between a plant’s environment and its genome, and metabolic profiling allows further investigation of the impact of plant phenology and biotic or abiotic stressors on plant phenotype [2,3,4]. Metabolic profiling is a technique that generates a unique chemical fingerprint for each sample and allows the identification of diverse set of plant secondary products (PSPs) in plant extracts [3,5]. PSPs are defined as metabolites of plant origin that are not related to housekeeping functions of an organism [3,6]. Metabolic profiling most often focuses on compounds of similar origin or chemical properties [7], and can be performed using both targeted and untargeted approaches [3]. Bioinformatics performed to interpret data generated by metabolic profiling results in an improved understanding of the plant’s response to varying environmental conditions at the biochemical, cellular and organismal level [4,8,9].

PSPs serve in plant defense against herbivores, microbes and competing plants [6]. PSPs may also play a crucial role in plant invasion as phytochemical weapons serving to inhibit organisms that are not evolutionarily adapted to detoxify or cope with such novel compounds [10,11]. In native ecosystems, herbivores, microbes and other plants have co-evolved and typically adapted to existing plant defense mechanisms by detoxification of toxic plant constituents [12]. Naive organisms in non-native ecosystems, however, did not co-evolve with endemic pathogens and herbivores and therefore the PSPs produced by invaders can be highly toxic to native species. In the introduced range, PSPs may therefore act as phytochemically unique “novel weapons” [10,11,13,14,15].

**Figure 1:**
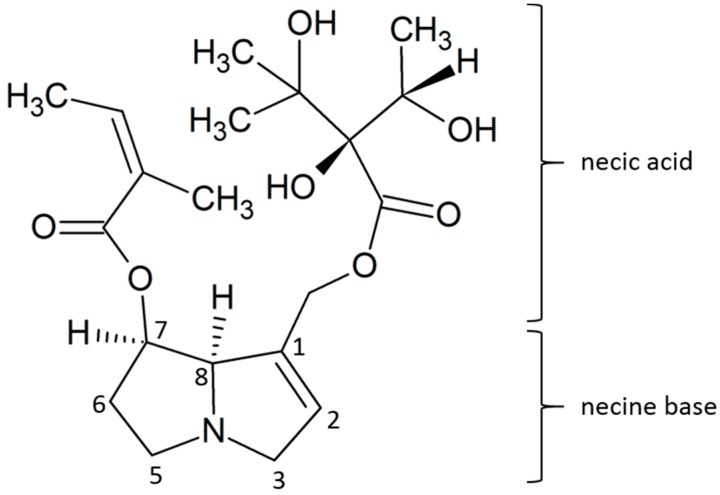
Structure of echimidine (image modified from [42]), the most abundant pyrrolizidine alkaloid (PA) in its *N*-oxide form in *Echium plantagineum* and *E. vulgare*. The unsaturated bond between positions 1 and 2 induces toxicity. Reversible oxidation of nitrogen at position four alters chemical properties. Side chains at positions 1 and 7 vary between analyzed alkaloids. A thorough review of PAs was published previously and is referred to for additional information [20].

Alkaloids are a family of complex PSPs that often contribute to plant constitutive defense mechanisms. Pyrrolizidine alkaloids (PAs), named for their characteristic double pyrrole ring structure (Figure 1), are interesting from an ecological perspective and are commonly found in the Asteraceae, Boraginaceae [16,17], Fabaceae [17] and Orchidaceae [18]. Although the chemistry of PAs has been well documented and over 350 structures are currently identified, their biosynthesis is not always well described. Our current understanding of their biosynthesis is generally based on several well-decribed examples [16,18]. Hartman and co-authors (1999) have presented one of the most complete reviews of the biosythesis of PAs in the Boraginaceae. The backbone of PA structure consists of a hydroxylated necine base forming two five-membered pyrrole rings with a bridgehead nitrogen atom at position 4 (Figure 1) [19]. The necine base originates from primary metabolites, specifically l-amino acids converted to homospermidine, the first specific PA precursor [12,16]. Necine bases are esterified with necic acids, usually derived from aliphatic amino acids that are transformed to C_7_ aliphathic acids [12].

PAs possess 1–2 unsaturated bonds which contribute to their toxicity; however, they are often stored in a non-toxic *N*-oxide (PANO) form in the plant or in an adapted insect. After foliar tissues are ingested by mammals and metabolized, oxidation can be reversible. PAs undergo a three-step metabolic process in hepatocytes to form tertiary alkaloids (free bases, pyrrols) that directly induce toxicity [17,18]. Pyrrole derivatives of PAs are highly reactive and can alkylate DNA and/or induce formation of DNA cross-linkages which interrupt DNA replication and incur mutations [20]. PAs may be hazardous, especially for non-ruminant animals, and the consumption of high quantities of foliage can lead to occasional death of cattle, horses and sheep [21]. Toxicity has also been observed in pigs [22] and rats when their diet was supplemented with toxic PAs [21]. Numerous citations have reported that the presence of PAs in the human diet can lead to severe health problems especially if consumed in large quantities [17,23,24,25]. PAs are associated with weak antileukemic and virustatic activity, carcinogenicity, embryotoxicity, hepatotoxicity, mutagenicity, and pneumotoxicity [19]. In addition to detrimental effects in mammalian systems, PAs can act as toxins to insect herbivores [26] as insect feeding deterrents [12,27] or stimulants for specialist insect herbivores [27].

*Echium plantagineum* L. and *E. vulgare* L. (Borgainaceae) produce a diverse suite of PSPs including toxic PAs, which accumulate primarily in foliar tissues [20,28,29], and root-produced naphthoquinones localized in living roots [28,30,31]. These species became exotic invaders in Australia shortly after their introduction in the 1800s. In their native range of the Iberian Peninsula, these species are uncommon and generally found in diverse mixtures with other plant flora [32,33]. In Australia, *E. plantagineum*, also known as Paterson’s curse or Salvation Jane, is an annual weed now naturalized over 30 M ha and dominates roadside plant communities, grazing lands and pastures [31,34,35]. *Echium vulgare*, or Viper’s bugloss, is a perennial and is limited to areas of higher rainfall and elevation in southern Australia where it typically does not have major impacts on Australian agriculture [33]. In direct contrast, the prevalence and toxicity of *E. plantagineum* have significantly impacted Australia’s textile and meat industries, causing an estimated annual loss of AUD $250 M and reduced pasture quality [36].

This study aimed to develop a useful platform for rapid and simultaneous profiling of PAs and PANOs in plant extracts using ultra high pressure liquid chromatography quadrupole time-of-flight (UHPLC-QTOF) mass spectrometry. This sensitive platform was used to study Australian populations of two related *Echium* species, more or less invasive, in both field and controlled glasshouse conditions. We compare the invasive status of these two species with respect to the production of PAs associated with plant defense and herbivore toxicity.

## 2. Results and Discussion

### 2.1. Profiling of Pyrrolizidine Alkaloids from Foliage

**Figure 2:**
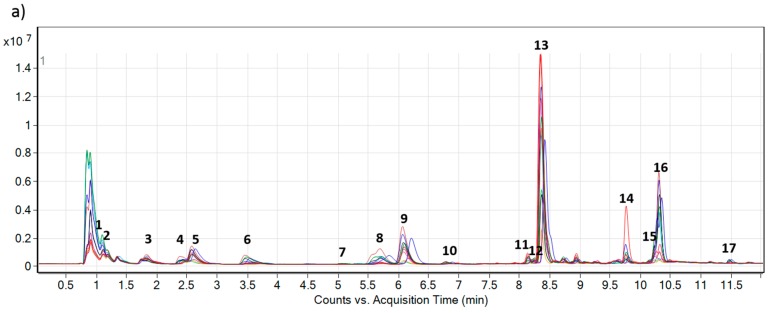

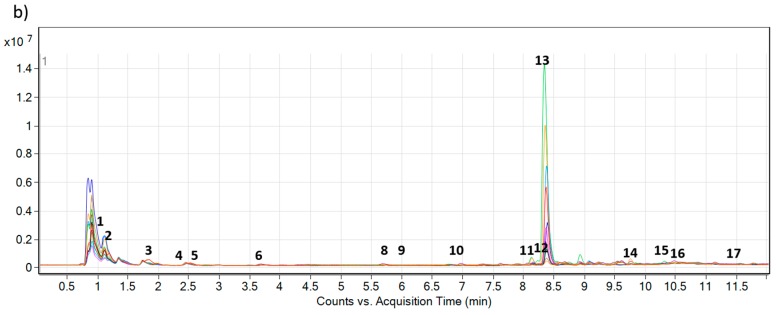
Overlaid total ion chromatograms (TIC) of 12 composite extracts (each represented by different color) of (**a**) *Echium plantagineum* and (**b**) *E. vulgare*, grown under identical controlled glasshouse conditions. Numbers 1–17 denote PAs and PANOs profiled in the study (Table 1).

High-speed, automated extraction of foliar tissue yielded high quality, uniform extracts that were further purified using solid phase extraction (SPE) to reduce sample matrix complexity for profiling alkaloids of interest. Concentrated samples contained alkaloids, specifically 20 more abundant compounds along with trace quantities of additional constituents. The UHPLC-QTOF MS method coupled to bioinformatics and statistical analysis along with the integration of published data on similar pyrrolizidine alkaloids allowed for annotation of 17 PAs and PANOs (Table 1; Figure 2) detected in extracts of foliar tissues of *E. plantagineum*. Earlier reports indicated the presence of five [37], twelve [20,28] or fourteen PAs in foliar tissues of this species [29]. The automated extraction process reduced leaf extraction time from 48 h [29] to 27 min. The use of ultra-high pressure liquid chromatography, coupled to sensitive and accurate LC/MS QTOF detection, allowed for improved separation of pyrrolizidine alkaloids, particularly stereoisomers or other closely related compounds (putative stereoisomers were referred to as compound A and B; Table 1; Figure 2). Seventeen PAs and PANOs (Table 1) were identified, mainly based on retention time (RT) and accurate mass (AM) as reported by Colegate and co-authors [29] who identified 14 related structures in 2005 (Table 1) in *E. plantagineum.* These authors also analysed PAs with HPLC/MS using authenticated standards in positive ionization mode.

Detection of PAs was performed in the past in different matrices, including honey, which required the use of advanced clean up procedures in addition to SPE. Several techniques were successfully used for analysis and identification of PAs including UV-Vis, NMR, HPLC [38], TLC, UHPLC-MS, and GC-MS [17,38]. Griffin *et al*. [39] developed a UHPLC ion trap MS method for analysis based on commercial standards available that allowed for identification of 11 PAs during a 30-min run. The constituents analysed by the present authors in plant extracts were less polar as they were not in the oxidized (PANO) form and typically eluted with higher concentrations of organic solvent (>20%) later in the gradient. In contrast, the method developed in this study allowed the annotation of 17 related PAs and PANOs of relatively high polarity during 18-min runs. Metabolic profiling of alkaloids using UHPLC-QTOF MS was also reported by Jaiswal *et al*. [5], who successfully identified 48 compounds including diterpene alkaloids in three separate plant species, and Zhu *et al*. [23], who developed the UHPLC-QTOF MS methodology for profiling of retronencine-type PAs in herbaceous plant extracts without corresponding standards.

In addition to 17 known PAs and PANOs, 18 additional PAs were characterized in *E. vulgare* in a previously reported study (Table S1) [20]. In our study, masses corresponding to several of the same PAs identified previously in *E. vulgare* were present in trace quantities or were completely absent in analysed extracts. Structural similarity and the presence of related compounds with identical masses (Table S1) made identification of all PAs relatively difficult. Structural confirmation of related PAs has previously required the use of other techniques for identification including NMR or GC-MS [20]. Although this study focused mainly on separation and profiling of compounds previously identified in *E. plantagineum*, structurally similar alkaloids were indeed present in both species, suggesting similarity in biosynthetic pathways among related *Echium* species. Interestingly, we detected ions of low abundance corresponding to the presence of intermedine, lycopsamine, acetyllycopsamine/intermedine, echiumine and 3’-acetylechiumine *N*-oxides in *E. vulgare* samples. These PANOs were not previously reported in *E. vulgare* (Table S1). The structural similarity of less well-described PAs observed in extracts of both *Echium* species further suggests that the LC/MS QTOF system we employed was highly sensitive, but this sensitivity does not negate the need for use of NMR for final structural confirmation of those alkaloids present in trace quantities.

When honey from Paterson’s curse was analysed, two additional PAs were reported using GC-MS analysis following the derivatization of purified honey extracts [40,41]. Constituents included 3’-acetyl derivatives of intermedine and lycopsamine [40,41]. Recently, the use of LC-MS plus targeted analysis with authenticated standards has resulted in identification of numerous compounds at trace levels; however, not all of these compounds were present in extracts of foliar tissues of *E. plantagineum* [28,29]. Non-targeted analysis of purified extracts of *E. plantagineum* resulted in the detection of acetylintermedine in leaf extracts. This method successfully allowed for rapid and simultaneous profiling of PAs of known origin but also allowed for additional data mining through untargeted analysis associated with the metabolism of PAs using a short run time of approximately 20 min.

### 2.2. Accumulation of PAs and PANOs in the Field Conditions

**Figure 3:**
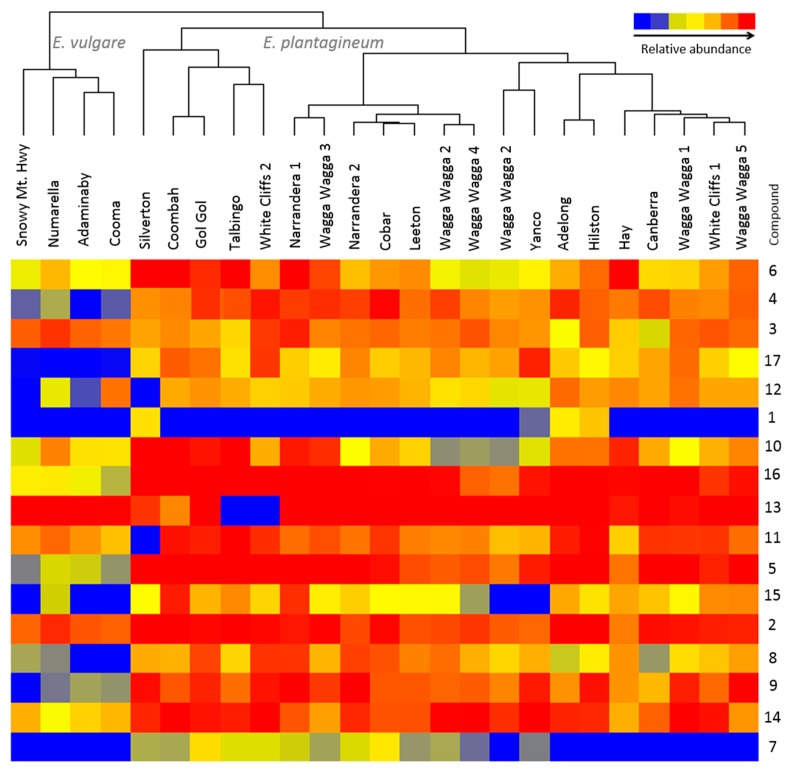
Variation in abundance of 17 pyrrolizidine alkaloids and their *N*-oxides (Table 1) in composite, field-collected samples of *Echium plantagineum* and *E. vulgare*. Hierarchical clustering algorithm and Euclidean distance metric were used on normalized abundance values. Dendrogram was generated using Mass Profiler Professional software (Agilent, Santa Clara, CA, USA).

The PAs and PANOs of geographically distinct populations of *E. plantaginuem* and *E. vulgare* surveyed and sampled across NSW indicated relatively little variation in production of PAs (Figure 3) among populations of *E. plantagineum* despite their collection from diverse climatic zones; in addition, some variation in plant morphology was observed among plant populations (*i.e*., plant height, leaf number, flower color). A longitudinal survey performed across the states of New South Wales, Victoria and Australian Capital Territory in Australia by Weston *et al*. [28] found significant positive correlation between latitude and production of PANOs. However, the correlation was not significant for elevation or longitude. In addition to 20 sampled populations of *E. plantagineum* in this experiment, we also included samples of *E. vulgare* that were collected from areas of higher elevation (Table S2). A survey of field sites in 2013–2014 showed considerable differences, with field samples separated into clusters by species (Figure 3). Profiling showed considerable variation in PAs and PANOs in *E. vulgare*, and leptanthine-*N*-oxide and 7-*O*-acetyllycopsamine/intermedine detected in *E. plantagineum* were not detected in *E. vulgare* samples. Only four field sites of *E. vulgare* infestation were identified and collected across New South Wales and Australian Capital Territory due to limited and/or sporadic establishment of this species in Australia [33]. Foliar extracts of both species were rich in echimidine and echiumine *N*-oxides, known for their potent toxicity to grazing herbivores. Least abundant compounds in both species included leptanthine-*N*-oxide and 7-*O*-acetyllycopsamine/intermedine, which were not detected in *E. vulgare*. Overall, *E. vulgare* produced up to four-fold lower total concentrations of PAs and PANOs than *E. plantagineum* (Figure 3).

**Table 1:** Chromatographic and spectrometric properties of related pyrrolizidine alkaloids (PAs) and their *N*-oxides (PANOs) profiled by ultra high pressure liquid chromatography quadrupole time-of-flight (UHPLC-QTOF) mass speS. Compounds were annotated based on *m/z* of molecular ions and retention time (RT) in relation to results published previously [29]. “A” and “B”—Denote potential isomers; AM—Accurate mass; * Denotes compounds which could be intermedine derivatives.

No.	Name	AM	Calculated [M + H]	Measured [M + H]	∆ppm	Approx. RT	Formula
1	Leptanthine-*N*-oxide	331.1631	332.1704	332.1701	0.90	1.08	C_15_H_25_NO_7_
2	Echimiplatine-*N*-oxide	331.1631	332.1704	332.1701	0.90	1.27	C_15_H_25_NO_7_
3	Uplandicine-*N*-oxide	373.1737	374.1809	374.1808	0.27	1.91	C_17_H_27_NO_8_
4	Intermedine-*N*-oxide	315.1682	316.1755	316.1754	0.32	2.35	C_15_H_25_NO_6_
5	Lycopsamine-*N*-oxide	315.1682	316.1755	316.1753	0.63	2.54	C_15_H_25_NO_6_
6	7-Angeloylretronencine-*N*-oxide	253.1314	254.1387	254.1384	1.18	3.42	C_13_H_19_NO_4_
7	7-*O*-Acetyllycopsamine *	341.1838	342.1911	342.1907	1.16	5.30	C_17_H_27_NO_6_
8	7-*O*-Acetyllycopsamine-*N*-oxide A *	357.1788	358.1860	358.1858	0.56	5.59	C_17_H_27_NO_7_
9	7-*O*-Acetyllycopsamine-*N*-oxide B *	357.1788	358.1860	358.1854	1.67	5.95	C_17_H_27_NO_7_
10	9-*O*-Angelylretronencine-*N*-oxide	253.1314	254.1387	254.1384	1.18	6.70	C_13_H_19_NO_4_
11	Echimidine-*N*-oxide A	413.2050	414.2122	414.2121	0.24	8.12	C_20_H_31_NO_8_
12	Echiuplatine-*N*-oxide	397.2101	398.2173	398.2170	0.75	8.18	C_20_H_31_NO_7_
13	Echimidine-*N*-oxide B	413.2050	414.2122	414.2120	0.48	8.32	C_20_H_31_NO_8_
14	3’-*O*-Acetylechimidine-*N*-oxide	455.2155	456.2228	456.2228	0.00	9.77	C_22_H_33_NO_9_
15	Echiumine-*N*-oxide A	397.2101	398.2173	398.2172	0.25	10.20	C_20_H_31_NO_7_
16	Echiumine-*N*-oxide B	397.2101	398.2173	398.2173	0.00	10.32	C_20_H_31_NO_7_
17	3’-*O*-Acetylechiumine-*N*-oxide	439.2206	440.2279	440.2276	0.68	11.53	C_22_H_33_NO_8_

A focused evaluation of leaf surface chemistry of these two species also showed similar results. *Echium plantaginuem* leaf dips typically contained more PAs and PANOs in greater abundance than did *E. vulgare;* leaf surface dips differed quantitatively and qualitatively, with echimidine being the most abundant PA detected in extracts of both species [43]. In our recent survey, field-collected plants of both species had low concentrations of lepthantine-*N*-oxide, which was previously found to be one of the most abundant alkaloids detected in field surveys across Australia [28]. Differences could be associated with variable weather conditions in the weeks prior to collection.

### 2.3. Growth Stage and Genotype-Dependent Accumulation of PAs and PANOs

Phenological stage often impacts the expression of complex biosynthetic pathways for PSPs. Increased production of phenolic compounds has been reported at the reproductive stage in multiple plant genera, and higher PSP abundance was often correlated with attraction of pollinators, an important ecological function [44]. The production of PAs and PANOs varies seasonally in *Senecio* [45] and also in *Echium,* with greater levels of naphthoquinones observed with increasing plant maturity [28]. In *E. plantagineum* and *E. vulgare*, root-produced naphthoquinones were produced within 48 hours following germination and were also significantly more abundant at plant maturity [31].

**Figure 4:**
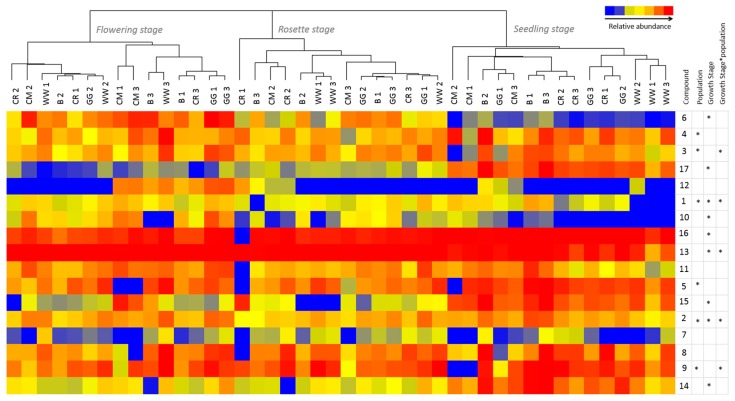
Relative abundance of 1–17 PANOs (Table 1) in extracts of five populations of *Echium plantagineum* harvested at three growth stages in controlled conditions experiment. Hierarchical clustering was performed using MPP Software on normalized values and Euclidean distance metric. *–denotes significance of population, growth stage and their interaction (repeated measures ANOVA; *p* < 0.05). Key to populations: B–Bendigo, CM—Coombah, CR—Cobar, GG—Gol Gol, WW—Wagga Wagga (numbers indicate block of replication).

**Figure 5:**
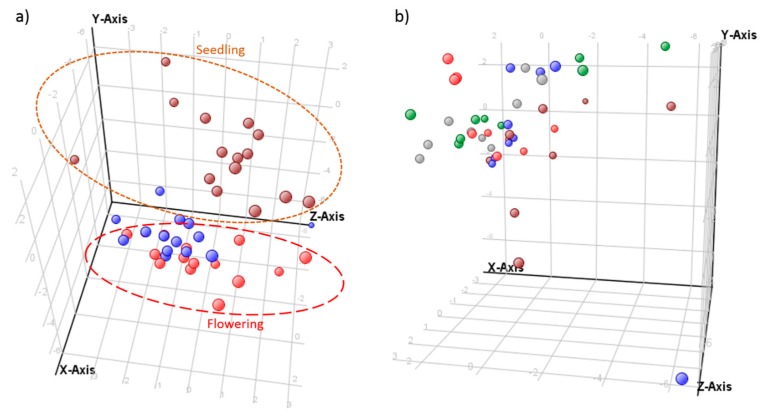
Principal component analysis (PCA) of controlled condition experiment, evaluated over five populations of *E. plantagineum*. (**a**) PCA of 17 PAs and PANOs and growth stage as independent variable. Component 1, 2 and 3 contribute separation by 23.39%, 21.04% and 12.45%, respectively. Discriminating metabolites for component 1 (PCA loadings > 0.3): 6, 11, 13 and for component 2 (PCA loadings > 0.3): 5, 8, 16, 17. Key to plant stages: seedling, brown; flowering plants, red and rosette, blue; (**b**) PCA of 17 PAs and PANOs with population as an independent variable. Component 1, 2 and 3 contribute to separation by 23.39%, 21.04% and 12.45%, respectively. Key to populations: Bendigo, red; Coombah, brown; Cobar, blue; Gol Gol, grey and Wagga Wagga, green.

After sampling at three different growth stages, PANOs were observed to be present in all investigated populations of *E. plantagineum* and limited variation between population and growth stage extracts was observed (Figure 4). The most abundant compounds at rosette and flowering stages were echimidine-*N*-oxide B (**13**) and echiumine-*N*-oxide B (**16**) while 3′-*O*-acetylechiumine-*N*-oxide (**17**) and 7-*O*-acetyllycopsamine/intermedine (**7**) were least abundant. Echiumine-related PAs and PANOs were significantly downregulated with increasing maturity in all studied populations. Similarly, 7-*O*-acetyllycopsamine/intermedine B (**9**), 3′-*O*-acetylechimidine (**14**) and lepthantine (**1**) *N*-oxides were significantly downregulated (*p* < 0.05) over time. However, the opposite trend was observed for retronencine-related PANOs, which were likely readily interconverted to other structures such as retronencine, the structural backbone of multiple PANOs in *Echium plantaginuem* [29]. Echimidine-*N*-oxide B (**13**) [40] was also significantly (*p* < 0.05) upregulated over time with plant maturation and was most abundant at rosette and flowering stage compared to the seedling stage in the majority of populations (Figure 4). Nine out of seventeen compounds varied with growth stage, and population × growth stage differences were clearly observed (Figure 5). Biosynthesis of PAs and PANOs was associated with phenological stage, with significant differences in chemical profiles observed between plants of flowering and seedling stage, with rosette stage intermediate. Although plant populations were generally similar in abundance of PAs, several individual compounds (**1**, **2**, **3**, **9** and **13**) responded differently over time with respect to population differences (Figure 4; Table S3).

A previous report on PAs in *Senecio jacobaea* (syn. *Jacobaea vulgaris*) showed elevated levels of PAs in the leaves at the flowering stage in comparison to the rosette stage, and floral extracts possessed the highest concentrations [17]. *Senecio* species biosynthesize PAs in their roots [12], and therefore PA foliar content may increase during transport to newly opened flowers. The location of biosynthesis of PAs in *E. plantagineum* has not been studied in great detail, but it has been suggested that site of production varies among species [46]. We have detected significant PA content in stem and leaf extracts, with considerable concentrations noted on leaf surfaces. We also detected relatively low concentrations of the three main alkaloids in root extracts as well, suggesting production or later translocation to living roots over time.

*Echium plantagineum* accumulated moderately high levels of PANOs within a week after germination. Accumulation of PAs of low abundance at the seedling stage, such as leptanthine-*N*-oxide (**1**) and 7-*O*-angeloylretronencine (**6**), increased over time (Figure 4). The biosynthesis of PAs occurred rapidly post germination, likely as a means to protect the plant and deter generalist herbivores [27]. PAs and PANOs potentially contribute to active plant defense by suppressing the feeding of insects and other herbivores as well as suppressing certain plants; together with antimicrobial naphthoquinones produced in the root periderm [31], they form a successful barrier and enhance plant resilience to pathogens and predators both above and below the ground.

Under a controlled glasshouse environment, variation in accumulation of PAs and PANOs was occasionally observed between populations (Figure 5). Six compounds were differentially expressed among populations. These included uplandicine-*N*-oxide (**3**), lycopsamine-*N*-oxide (**5**) and 7-*O*-acetyllycopsamine/intermedine-*N*-oxide B (**9**) which were significantly downregulated (*p* < 0.05) in the Coombah population (Figure 4; Table S3). Interestingly, the Coombah population also exhibited reduced shikonin biosynthesis in its roots in a separate study and these differences were attributed to possible genetic differences in PSP biosynthesis [31]. Leptanthine-*N*-oxide (**1**) was significantly downregulated in seedlings of the Wagga Wagga population.

### 2.4. Comparative Profiling of Echium Plantagineum and E. Vulgare

Metabolic profiles of both *Echium* species in Australia were recently compared with respect to production of PSPs, specifically quinone-containing shikonins in the roots. These studies revealed that shikonin biosynthesis is highly conserved across species, with limited differences in metabolic profiles [31]. Five populations of each species were used in this evaluation of PAs and PANOs. Plants were harvested and extracted after 6–8 weeks at the rosette stage and at 27–29 weeks when *E. plantagineum* was fully flowering; interestingly, we observed in this study that *E. vulgare* did not flower and remained in the vegetative growth stage, likely due to its perennial growth habit and lack of exposure to vernalizing conditions in the greenhouse [33].

**Figure 6:**
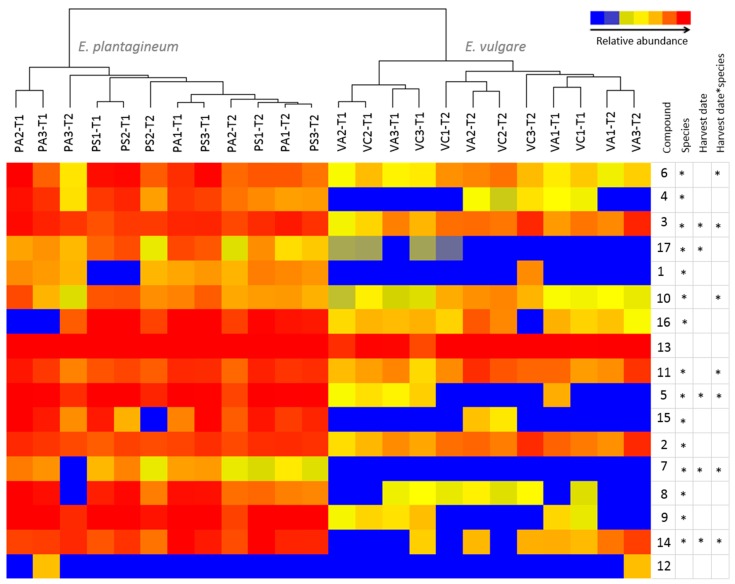
Comparison of abundance of selected pyrrolizidine alkaloids and their *N*-oxides in *Echium plantagineum* and *E. vulgare* plants grown in uniform conditions in the glasshouse. Hierarchical clustering algorithm and Euclidean distance metric were used on normalized abundance values. Dendrogram was generated using MPP software. *-denotes significance of population, harvest date and their interaction (repeated measures ANOVA, *p* < 0.05). Key to populations (at top of diagram): PA, *E. plantagineum* from Adaminaby; PS, *E. plantagineum* from Silverton; VA, *E. vulgare* from Adelong; VC, *E. vulgare* from Cooma. Key to plant stages: T1, first harvest; T2, second harvest.

Metabolic profiling revealed that 15 out of 17 PAs or PANOs were significantly downregulated in *E. vulgare* (*p* < 0.05) (Figure 6; Table S4); however, 7-*O*acetyllycopsamine/intermedine (**7**) and uplandicine (**3**), lycopsamine (**5**), 7-angeloylretronencine (**6**), 9-*O*-angelylretronencine (**10**), echimidine A (**11**) and 3’-*O*-acetylechimidine (**14**) *N*-oxides were expressed differentially in both species over time. Echimidine-*N*-oxide B (**13**) was the most abundant PANO in both species, and has been described as responsible for acute alkaloid toxicity in rats [40]. Echimidine was previously found in high quantities in *E. vulgare* and *E. setosum* [47]. Rare or less common PAs observed included 7-*O*-acetyllycopsamine/intermedine (**7**) > leptanthine-*N*-oxide (**1**) > echiuplatine-*N*-oxide (**12**). 7-*O*-acetyllycopsamine/intermedine (**7**) was absent in *E. vulgare* while lepthantine-*N*-oxide (**1**) was found in only one sample of this species. Echiuplatine-*N*-oxide (**12**) was uncommon and was identified in one sample of each species of plants grown in controlled conditions (Figure 6). Variation in in phenology associated with growth habit may be important in regulating PA expression.

**Figure 7:**
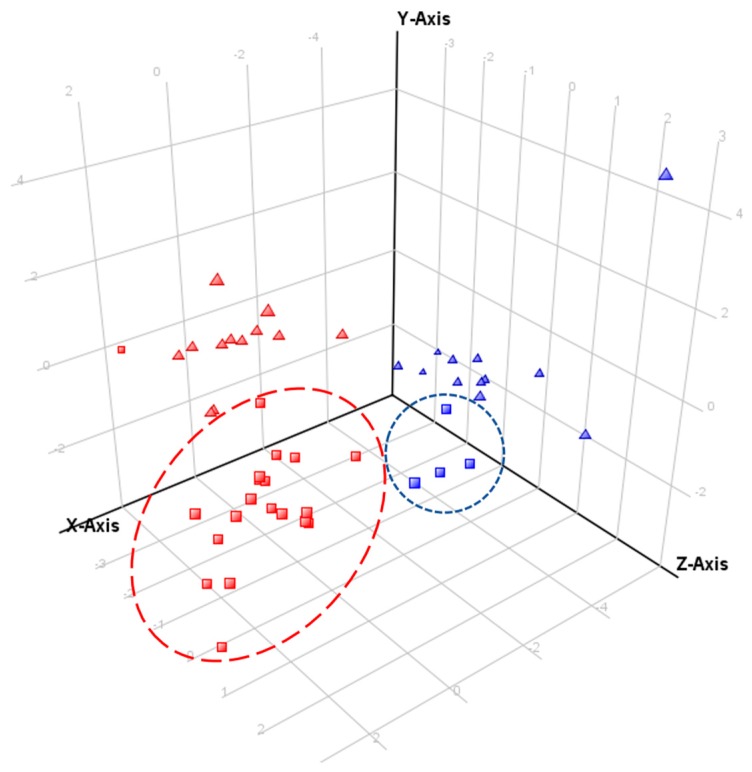
Principal component analysis (PCA) of samples of both species from the field experiment and controlled conditions experiment. PCA included 17 PANOs and growth stage as independent variable. Component 1, 2 and 3 contribute to separation by 41.83%, 11.58% and 9.75%, respectively. Discriminating metabolites for component 1 (PCA loadings ≥ 0.3): 5, 6, 17 and for component 2 (PCA loadings > 0.4): 1. Key to symbols: *Echium plantagineum*, red; *E. vulgare*, blue; field survey samples, squares; glasshouse samples, triangles. Outlying field sample of *E. plantagineum* was collected in Silverton (Table S2).

Metabolic profiles of both species were compared using a comprehensive personal compound database (Table 1) based on accurate mass (AM) and retention time (RT) of compounds earlier reported in *E. plantagineum*. Several compounds profiled in this study were found with high mass accuracy in both species and were not previously reported in *E. vulgare* extracts. However, as previously mentioned, the complexity and structural similarity of many PAs and PANOs renders positive identification of metabolites a complex task, particularly for those present in trace quantities. The concentration of PANOs and PAs in *E. vulgare* was significantly lower in glasshouse-grown plants when both species were produced under uniform conditions (Figure 2 and Figure 6). *Echium vulgare* produced more PANOs under field conditions, but their abundance was lower than in *E. plantagineum* (Figure 7).

The three most abundant PANOs in *E. vulgare* under field and glasshouse conditions were: echimidine-*N*-oxide B (**13**) > echimiplatine-*N*-oxide (**2**) > uplandicine-*N*-oxide (**3**). In contrast, *E. plantagineum* field-collected plants produced lycopsamine-*N*-oxide (**5**) > echimidine-*N*-oxide B (**13**) > echiumine-*N*-oxide B (**16**) whereas glasshouse-grown plants produced echimidine-*N*-oxide B (**13**) > echiumine-*N*-oxide B (**16**) > 7-*O*-acetyllycopsamine/intermedine-*N*-oxide B (**9**). Molecular ions of PAs and PANOs previously reported in *E. vulgare* [20] were also investigated (Table S1) in field and glasshouse plant extracts. Ions of 31 listed PAs and PANOs were identified with high accuracy; however, previously reported molecular ions of 7-angeloyl-9-(2-methylbutryl)-retronecine, 7-tigloyl-9-(2-methylbutryl)-retronecine, 7-*O*-acetyllycopsamine/intermedine, asperumine and vulgarine [20] were not observed.

Additionally, the peak of *m/z* 416.2276 at 8.7 min (Figure 2) found in *E. plantaginuem* samples (generated formulae: C_20_H_33_NO_8_) had a similar accurate mass to the *N*-oxide of canescine/canescenine (*m/z* 399.2257). In addition to abundant *N*-oxides, untargeted analysis and comparison to Metlin database (Agilent, Santa Clara, CA, USA) allowed for putative annotation of several PAs. Annotated compounds included acetylintermedine (*m/z* 342.1901; 5.67 min) found in *E. plantaginuem* samples only and reported in literature previously [20], less abundant symlandine (*m/z* 382.2224; 10.27 min) in *E. vulgare* and also petasitenin (*m/z* 382.186; 8.85 min) in *E. plantagineum*.

Results suggested that qualitative and quantitative differences between the *Echium* species in terms of alkaloid production may be of biological importance with respect to their invasion success. Consistently elevated levels of PAs and PANOs observed in *E. plantagineum* may potentially deter generalist herbivores more successfully and thereby impact invasion success of this species in its non-native range.

## 3. Experimental Section

### 3.1. Chemicals and Standards

HPLC grade solvents were used in this study and included acetonitrile (Hipersolv, Tingalpa, Australia), water (Merck, Darmstadt, Germany) and methanol (Burdick & Jackson, Muskegon, MI, USA). Ammonium hydroxide (Merck) and sulfuric acid (ThermoScientific, Univar, Australia) were diluted in methanol and water to concentrations of 0.72 M and 0.05 M, respectively. Formic acid, >99% purity (Sigma, Castle Hill, Australia), was used to acidify the mobile phase. Crotaline, a well-studied pyrrolizidine alkaloid (SigmaAldrich, Castle Hill, Australia), was used as a known standard for methods development.

### 3.2. Instrumentation

Metabolic profiling of filtered shoot extracts was performed using an Agilent 1290 Infinity UHPLC system equipped with quaternary pump, diode array detector, degasser, temperature controlled column and cooled autosampler compartments, coupled to an Agilent 6530 quadrupole time-of-flight (Q-TOF) mass spectrometer with Agilent Dual Jet Stream ESI ionization source (Agilent Technologies, Mulgrave, Australia).

### 3.3. Collection of Field-Grown Plant Material

Plant samples were collected from 20 geographically distinct field sites for *E. plantagineum* across New South Wales (NSW) and the Australian Capital Territory (ACT) in Australia between 2013 and 2015 (Table S2). In contrast, due to its limited range in Australia, *E. vulgare* was collected from only four geographically distinct field sites in NSW and the ACT during the same period. Prior to extraction, a composite sample of five leaves per plant population was pooled from each site to minimize plant-to-plant-to-plant variation. Seed from each population was collected from 5 to 10 individual plants and stored in the laboratory in the dark at ambient temperature prior to seeding in the glasshouse.

### 3.4. Glasshouse Grown Plant Material

#### 3.4.1. Germination and Plant Growth Conditions

Field-collected seed lots from populations of both species were germinated over a seven days on moist filter paper in an incubator with 12:12 (light:dark) light and 25:15 °C (light:dark) temperature regimes. One set of seedlings was retained for later extraction while the remainder was transplanted into 1.5-L pots containing a mixture of potting mix and sand (6:4, respectively). Plants were generally grown in the glasshouse under natural light conditions with the exception of Experiment 1, where daylength was extended to a minimum of 14 h in winter at Charles Sturt University (Wagga Wagga, NSW, Australia) under an alternating temperature regime ranging from 25 ± 4 °C day and 17 ± 3 °C night. Plants were watered as needed and fertilized fortnightly with 200 mL of Aquasol soluble fertilizer (Yates, Australia) prepared according to the manufacturer’s instructions. Populations of *Echium* spp. were grown in a randomized block design with three blocks in each experiment.

#### 3.4.2. Echium Plantagineum Phenological Study—Experiment 1

Seed collected from five separate geographically distinct *E. plantagineum* field populations was separately germinated and reared to generate 12 plants per five populations with 60 total plants per replicate and 180 total plants per experiment (Table S5). Four plants per replicate per population were harvested at each of three different maturity stages: 1-week-old seedlings, 7- to 9-week-old rosettes and 11- to 14-week-old flowering plants (flowering = over 80% with open inflorescences). At harvest time, replicates were harvested by block and extracted sequentially starting with Replicate 1 which required five full days to complete tissue processing, followed by extraction of Replicate 2, and finally Replicate 3. Tissue of four plants derived from the same population was pooled for extraction to yield one sample per replicate per time point, each sample being a composite of the mature leaves of four individuals to minimize plant/plant variation. This experiment was performed from 29 July until 17 December 2013 with minimum of 14 light hours obtained using additional lighting (400 µmoles/m^2^/s).

#### 3.4.3. Comparative Profiling of Echium Species—Experiment 2

Two populations of *E. plantagineum* and *E. vulgare* were cultivated in an additional randomized block design experiment performed in a similar manner to that of Experiment 1 (Table S6). Eight plants × two populations × two species were raised per replicate, generating 32 plants per replicate with a total of 96 plant per experiment. Plants were harvested at two maturity stages: before and after flowering of *E. plantagineum* (*E. vulgare* never flowered under glasshouse conditions). The tissue of four plants derived from the same population was pooled for extraction to yield one sample per replicate per time point, each sample being a composite of the mature leaves of four individuals, to minimize plant/plant variation. This experiment was performed from 5 March until 2 October 2014.

### 3.5. Extraction

#### 3.5.1. Foliar Tissue Extraction

Leaves (4.00 g) were chopped and extracted with methanol (40 mL) under pressure (100 bar) at 35 °C using a Büchi Speed Extractor (Model E-916, Büchi Corporation, Flawil, Switzerland) in two consecutive cycles. Samples were dried using a rotary evaporator (Multivapour P-6, Büchi) at 35 °C and reconstituted to 21 mL in methanol and stored at 4 °C.

#### 3.5.2. Solid Phase Extraction (SPE)

Solid phase extraction (SPE) was performed to minimize matrix complexity of crude leaf extracts and enhance the abundance of pyrrolizidine alkaloids under evaluation as per Colegate *et al*. (2005). Leaf extracts were purified using Strata SCX, 500 mg, 3 mL SPE cartridges (Phenomenex, Torrance, CA, USA) [28,29]. Five mL of extracts were dried under N_2_ gas at 35 °C. Samples were then resuspended in 1 mL of 0.05 M sulphuric acid and vortexed, of which 100 µL was loaded on the column. Elution was performed with 6 mL of 0.72 M ammonia/methanol solution. Eluent was evaporated under N_2_ gas in a dry block heater and residue was resuspended in 1 mL of methanol.

### 3.6. UHPLC-MS Analysis

One µL of extracts was injected and separated using a C_18_ Poroshell column (2.1 mm × 100 mm, 2.7 µm particle size) (Agilent Technologies, Santa Clara, CA, USA), preceded by an SB-C_8_ guard column (2.1 mm × 12.5 mm, 5 µm particle size) (Agilent Technologies) maintained at 25 °C. The mobile phase consisted of solvent A (water, 0.1% formic acid) and solvent B (95% acetonitrile, 0.1% formic acid) and the flow rate was set to 0.3 mL/min. The column was equilibrated for 40 min prior to first injection. Separation was obtained using a gradient of solvents beginning with high polarity, 10% B for 2 min. The gradient increased to 50% B over 10 min. At 12.6 min, the mobile phase returned to 10% B and continued until 18 min. The column was flushed with 100% B for 20 min ca. every 10 samples. The Q-TOF was calibrated in the positive ion mode with nebulizer gas set at 35 psig, capillary voltage at 3500 V and fragmentor voltage at 135 V. Nitrogen was used as the drying gas at 250 °C at a flow of 9 L/min. Sheath gas was supplied at 10 L/min at 400 °C. Data were collected in positive ion, scan mode in extended dynamic range (2 GHz).

### 3.7. Data Analysis

#### 3.7.1. UHPLC-MS Data Analysis

Mass spectrometer data were characterized manually in Mass Hunter Workstation Qualitative Software version B06.00 Qualitative Analysis (Agilent Technologies) and compared to results obtained by Colegate *et al*. [29] and Weston *et al*. [28]. Retention time (RT) of known compounds was adjusted and included in a personal compound database and library (PCDL) software (version B04.00, Agilent Technologies, Santa Clara, CA, USA). PCDL was used to screen sets of data (following the adjustment of RT in PCDL) using Find by Formula (FbF) algorithm in MassHunter Workstation software (version B06.00 Qualitative Analysis, Agilent Technologies). Processed data were evaluated manually to eliminate misidentifications caused by similarity of RT and AM of the compounds. Results were later exported to compound exchange file (CEF) format and analyzed and visualized in Mass Profiler Professional (MPP) software (Agilent Technologies) using principal component analysis and hierarchical clustering algorithm. Profinder software (Agilent, Santa Clara, CA, USA) in batch recursive workflow was used to find molecular features. MPP was used to select entities represented by at least two ions, present in more than one sample and peak height > 5000 counts. One-hundred and seventy-four final entities were compared to the Metlin Metabolites database (Agilent Technologies).

#### 3.7.2. Statistical Analysis

Repeated measures analysis of variance was performed in Statistix 9 (Analytical Software, Talahassee, FL, USA) for replicated experiments that included time variable. Compound abundance data was log transformed prior to statistical analyses. Standard error of the means was used to show the variability of the abundance. Additional analysis was performed in Microsoft Excel.

## 4. Conclusions

PSPs may contribute to plant invasion success by enhancing mechanisms of plant defense or competitiveness [13]. Plants introduced to novel environments may have the advantage of escape from their natural biocontrol agents [48] or competitors and in some cases may easily adapt to novel environments as is the case of *E. plantagineum* in contrast to *E. vulgare* [49]. Metabolic profiling of plant defense metabolites revealed significant upregulation of many toxic metabolites, specifically PAs and PANOs, in the highly invasive species, *E. plantagineum*. This species has spread across 30 M ha of acreage in Australia, while *E. vulgare* remains confined to small regions of higher elevation across NSW and the ACT. Interestingly, *E. plantagineum* accumulated significantly higher levels of PAs and PANOs in both field and glasshouse conditions in contrast to the much less invasive *E. vulgare*. A UHPLC-QTOF MS platform was successfully employed to compare metabolic profiles of PAs in these congeneric species, with a high level of sensitivity and a relatively short run time of 18 min. Although more PA ions were detected in *E. vulgare* in contrast to *E. plantagineum* samples, structural elucidation of all compounds detected (>35 alkaloids) was not attempted. Biosynthesis of PAs and PANOS in both *Echium* species is likely similar; however, the diversity of related metabolites was higher in *E. vulgare* extracts, while relative abundance of toxic constituents was significantly lower. This may be due to enhanced catabolism or metabolic turnover of certain molecules in perennial *versus* annual species. Interestingly, echimidine *N*-oxides were the most abundant in extracts of both species, which suggests that the biosynthetic pathway for PA production is likely to be conserved among species. Echimidine-type alkaloids are also responsible for the majority of toxicity outbreaks in grazing herbivores. Phenological stage also impacted production as PAs were generally more abundant over time with increased plant maturity. While alkaloids often function in plant defense, we propose that most insects in Australia do not have the ability to detoxify PAs and PANOs, and PAs may play important roles as novel weapons particularly in non-native ranges of expansion. Studies performed in our laboratory indicated the increase of production of PAs in *E. plantagineum* when above-ground plant parts were grazed by a generalist herbivore. However, no significant response was noted to feeding by the specialist herbivore *Mogulones larvatus* [50]. These studies suggest that *E. plantagineum* may become increasingly toxic when ingested by non-adapted organisms. With the spread of this species over time, and successful adaptation to increasingly challenging climatic conditions, including drought and high temperatures, the possibility of increasing toxicity related to enhanced production of PAs due to upregulation of biosynthesis is an important consideration, especially as it relates to enhanced toxicity to grazing herbivores. Past studies have shown strong correlation of production of PAs with warm and dry climates. Therefore, PAs may play an important role as novel weapons in the range expansion of this noxious species across Australia, particularly in a changing climate.

## Supplementary Materials

Supplementary materials can be found at http://www.mdpi.com/1422-0067/16/11/25979/s1.
