# Supplementary material for: Metabolic Profiling of Pyrrolizidine Alkaloids in Foliage of Two Echium spp. Invaders in Australia—A Case of Novel Weapons?
Source: Int J Mol Sci. 2015 Nov 6;16(11):26721–37. doi: 10.3390/ijms161125979 (PMC4661838; doi:10.3390/ijms161125979)
Supplement: Supplementary file 1 [file ijms-16-25979-s001.pdf]

International Journal of
Molecular Sciences

MDPI

Supplementary Information

Table S1. Mass spectrometric and chemical properties of selected PANOs and PAs, reported in literature and/or profiled in the study. Accurate masses of molecular ions that are unique for a compound are highlighted in blue. Remaining masses are repeatable among multiple compounds making them difficult to identify. PCDL—personal database and library.

No.	Pyrrolizidine Alkaloid	Formula	*M*w	[M + H]	[N-oxide + H]	Reported in *E. vulgare* [20]	Included in PCDL
1	3′-Acetylechimidine	$C_{22}H_{33}NO_8$	439.2206	440.2279	456.2228	Yes	Yes
2	3′-Acetylheliosupine	$C_{22}H_{33}NO_8$	439.2206	440.2279	456.2228	Yes	No
3	3′-*O*-Acetylechiumine	$C_{22}H_{33}NO_7$	423.2257	424.233	440.2279	No	Yes
4	5′-Acetylechimidine	$C_{22}H_{33}NO_8$	439.2206	440.2279	456.2228	Yes	No
5	7-(2-methylbutyryl) retronecine	$C_{13}H_{21}NO_3$	239.1521	240.1594	256.1543	Yes	No
6	7-Angeloyl-9-(2,3-di methylbutryl) retronecine	$C_{19}H_{29}NO_4$	335.2097	336.2169	352.2118	Yes	No
7	7-Angeloyl-9-(2-methylbutryl) retronecine	$C_{18}H_{27}NO_4$	321.194	322.2013	338.1962	Yes	No
8	7-Angeloylretronecine	$C_{13}H_{19}NO_3$	237.1365	238.1438	254.1387	Yes	Yes
9	7-*O*-Acetyllycopsamine	$C_{17}H_{27}NO_5$	325.1889	326.1962	342.1911	No	Yes
10	7-*O*-Acetyllycopsamine/intermedine A	$C_{17}H_{27}NO_6$	341.1838	342.1911	358.186	No	Yes
11	7-*O*-Acetyllycopsamine/intermedine B	$C_{17}H_{27}NO_6$	341.1838	342.1911	358.186	No	Yes
12	7-*O*-acetylvulgarine	$C_{22}H_{33}NO_8$	439.2206	440.2279	456.2228	Yes	No
13	7-Tigloyl-9-(2, 3-dihydroxybutryl)retronecine	$C_{17}H_{25}NO_6$	339.1682	340.1755	356.1704	Yes	No
14	7-Tigloyl-9-(2-methylbutryl) retronecine	$C_{18}H_{27}NO_4$	321.194	322.2013	338.1962	Yes	No
15	7-Tigloylretronecine	$C_{13}H_{19}NO_3$	237.1365	238.1438	254.1387	Yes	No
16	9-(2-methylbutyryl) retronecine	$C_{13}H_{21}NO_3$	239.1521	240.1594	256.1543	Yes	No
17	9-Angeloylretronecine	$C_{13}H_{19}NO_3$	237.1365	238.1438	254.1387	Yes	Yes
18	9-Tigloylretronecine	$C_{13}H_{19}NO_3$	237.1365	238.1438	254.1387	Yes	No
19	Asperumine	$C_{18}H_{25}NO_4$	319.1784	320.1856	336.1805	Yes	No
20	Echihumiline	$C_{20}H_{31}NO_7$	397.2101	398.2173	414.2122	Yes	No
21	Echimidine A	$C_{20}H_{31}NO_7$	397.2101	398.2173	414.2122	Yes	Yes
22	Echimidine B	$C_{20}H_{31}NO_7$	397.2101	398.2173	414.2122	Yes	Yes
23	Echimiplantine	$C_{15}H_{25}NO_6$	315.1682	316.1755	332.1704	Yes	Yes
24	Echinatine	$C_{15}H_{25}NO_5$	299.1733	300.1805	316.1755	Yes	No
25	Echiumine A	$C_{20}H_{31}NO_6$	381.2151	382.2224	398.2173	No	Yes
26	Echiumine B	$C_{20}H_{31}NO_6$	381.2151	382.2224	398.2173	No	Yes
27	Echiuplantine	$C_{20}H_{31}NO_6$	381.2151	382.2224	398.2173	Yes	Yes
28	Echivulgarine	$C_{25}H_{38}NO_8$	480.2597	481.267	497.2547	Yes	No
29	Heliosupine	$C_{20}H_{31}NO_7$	397.2101	398.2173	414.2122	Yes	No
30	Intermedine	$C_{15}H_{25}NO_5$	299.1733	300.1805	316.1755	No	Yes

Table S1. *Cont.*

No.	Pyrrolizidine Alkaloid	Formula	M_W	[M + H]	[N-oxide + H]	Reported in *E. vulgare* [20]	Included in PCDL
31	Leptanthine	$C_{15}H_{25}NO_6$	315.1682	316.1755	332.1704	Yes	Yes
32	Lycopsamine	$C_{15}H_{25}NO_5$	299.1733	300.1805	316.1755	No	Yes
33	Retronecine	$C_8H_{13}NO_2$	155.0946	156.1019	172.0968	Yes	No
34	Uplandicine	$C_{17}H_{27}NO_7$	357.1788	358.186	374.1809	Yes	Yes
35	Vulgarine	$C_{11}H_{18}NO_4$	228.1236	229.1309	254.1258	Yes	No

Table S2. GPS data of surveyed locations of *Echium plantaginuem* and *E. vulgare*.

Location Name	Species	Latitude	Longitude	Elevation [m]
Adaminaby	*E. vulgare*	−35.935290	148.663425	1196
Adelong	*E. plantagineum*	−35.197103	147.885227	346
Canberra	*E. plantagineum*	−35.268581	149.164344	737
Cobar	*E. plantagineum*	−31.577727	145.136038	230
Cooma	*E. vulgare*	−36.245625	149.027277	927
Coombah	*E. plantagineum*	−32.982600	141.628500	40
Gol Gol	*E. plantagineum*	−34.194507	142.238537	57
Hay	*E. plantagineum*	−34.497130	144.831042	99
Hillston	*E. plantagineum*	−32.991505	145.900375	166
Leeton	*E. plantagineum*	−34.533012	146.408958	151
Narrandera 1	*E. plantagineum*	−34.754452	146.537492	155
Narrandera 2	*E. plantagineum*	−34.755983	146.523143	167
Numeralla	*E. vulgare*	−36.172651	149.348837	738
Silverton	*E. plantagineum*	−31.882900	141.228100	258
Snowy Mt. Hwy	*E. vulgare*	−35.935290	148.663425	1196
Talbingo	*E. plantagineum*	−35.407947	148.290573	522
Wagga 1	*E. plantagineum*	−35.051878	147.348191	185
Wagga 2	*E. plantagineum*	−35.051878	147.348191	245
Wagga 3	*E. plantagineum*	−35.051878	147.348191	289
Wagga 4	*E. plantagineum*	−35.051878	147.348191	228

Table S2. *Cont.*

Location Name	Species	Latitude	Longitude	Elevation [m]
Wagga 5	*E. plantagineum*	−35.052000	147.347000	272
White Cliffs 1	*E. plantagineum*	−31.017915	143.045348	138
White Cliffs 1	*E. plantagineum*	−31.017915	143.045348	138
White Cliffs 2	*E. plantagineum*	−30.850300	143.088890	163
Yanco	*E. plantagineum*	−34.615736	146.422218	158

Table S3. Results of repeated measures ANOVA performed in Statistix 9 software for the data collected in controlled conditions experiment with 5 populations of *Echium plantaginuem* harvested at 3 time points. Numbers 1–17 denote pyrrolizidine alkaloids and their N-oxides profiled in the study (Table 1).

Compound	Source GH1	df	Means Square	*F* Value	*p* Value
Leptanthine-*N*-oxide (1)	Population	4	6.02692	8.76	0.0026
	Error Population × Replicates	10	0.68802	-	-
	Time	2	6.63138	7.23	0.0046
	Population × Time	8	8.99772	9.80	0.0000
	Error Population × Replicates × Time	19	0.91780	-	-
Echimiplatine-*N*-oxide (2)	Population	4	0.18159	8.55	0.0029
	Error Population × Replicates	10	0.02125	-	-
	Time	2	0.21417	6.59	0.0067
	Population × Time	8	0.23682	7.29	0.0002
	Error Population × Replicates × Time	19	0.03248	-	-
Uplandicine-*N*-oxide (3)	Population	4	2.11474	3.74	0.0412
	Error Population × Replicates	10	0.56535	-	-
	Time	2	1.47490	1.98	0.1659
	Population × Time	8	2.15739	2.89	0.0274
	Error Population × Replicates × Time	19	0.74599	-	-

Table S3. *Cont.*

Compound	Source GH1	df	Means Square	*F* Value	*p* Value
Intermedine-*N*-oxide (4)	Population	4	0.72309	3.85	0.0380
	Error Population × Replicates	10	0.18759	-	-
	Time	2	0.72836	3.44	0.0532
	Population × Time	8	0.26502	1.25	0.3246
	Error Population × Replicates × Time	19	4.02642	-	-
Lycopsamine-*N*-oxide (5)	Population	4	10.3847	9.90	0.0017
	Error Population × Replicates	10	1.0486	-	-
	Time	2	2.3370	0.52	0.6015
	Population × Time	8	3.6937	0.83	0.5909
	Error Population × Replicates × Time	19	4.4761	-	-
7-Angeloylretronencine-*N*-oxide (6)	Population	4	2.1628	1.40	0.3022
	Error Population × Replicates	10	1.5437	-	-
	Time	2	31.1116	33.93	0.0000
	Population × Time	8	0.9787	1.07	0.4248
	Error Population × Replicates × Time	19	0.9169	-	-
7-*O*-Acetyllycopsamine\intermedine (7)	Population	4	15.0375	3.21	0.0613
	Error Population × Replicates	10	4.6844	-	-
	Time	2	3.0802	1.00	0.3859
	Population × Time	8	3.7313	1.21	0.3433
	Error Population × Replicates × Time	19	3.0756	-	-
7-*O*-Acetyllycopsamine\intermedine-*N*-oxide A (8)	Population	4	2.84120	1.72	0.2219
	Error Population × Replicates	10	1.65267	-	-
	Time	2	1.58873	2.25	0.1322
	Population × Time	8	1.21906	1.73	0.1556
	Error Population × Replicates × Time	19	0.70458	-	-
7-*O*-Acetyllycopsamine\intermedine-*N*-oxide B (9)	Population	4	6.76368	4.84	0.0196
	Error Population × Replicates	10	1.39634	-	-
	Time	2	0.69941	1.57	0.2337
	Population × Time	8	1.84337	4.14	0.0052
	Error Population × Replicates × Time	19	0.44529	-	-

Table S3. *Cont.*

Compound	Source GH1	df	Means Square	*F* Value	*p* Value
9-*O*-Angelylretronencine-*N*-oxide (10)	Population	4	4.4088	1.46	0.2840
	Error Population × Replicates	10	3.0109	-	-
	Time	2	36.1672	8.78	0.0020
	Population × Time	8	4.0541	0.98	0.4776
	Error Population × Replicates × Time	19	4.1179	-	-
Echimidine-*N*-oxide A (11)	Population	4	2.37800	2.26	0.1351
	Error Population × Replicates	10	1.05330	-	-
	Time	2	1.71373	1.82	0.1888
	Population × Time	8	1.07352	1.14	0.3816
	Error Population × Replicates × Time	19	0.94045	-	-
Echiuplatine-N-oxide (12)	Population	4	3.5336	0.61	0.6619
	Error Population × Replicates	10	5.7488	-	-
	Time	2	23.2923	1.67	0.2139
	Population × Time	8	6.1827	0.44	0.8792
	Error Population × Replicates × Time	19	13.9091	-	-
Echimidine-*N*-oxide B (13)	Population	4	0.16444	1.59	0.2509
	Error Population × Replicates	10	0.10334	-	-
	Time	2	1.11181	11.90	0.0004
	Population × Time	8	0.28129	3.01	0.0232
	Error Population × Replicates × Time	19	0.09346	-	-
3′-*O*-Acetylechimidine-*N*-oxide (14)	Population	4	1.49266	2.70	0.0921
	Error Population × Replicates	10	0.55219	-	-
	Time	2	8.77273	9.37	0.0015
	Population × Time	8	1.31951	1.41	0.2554
	Error Population × Replicates × Time	19	0.93663	-	-
Echiumine-*N*-oxide A (15)	Population	4	3.7318	1.47	0.2837
	Error Population × Replicates	10	2.5469	-	-
	Time	2	23.5189	10.20	0.0010
	Population × Time	8	4.2476	1.84	0.1307
	Error Population × Replicates × Time	19	2.3049	-	-

Table S3. *Cont.*

Compound	Source GH1	df	Means Square	*F* Value	*p* Value
Echiumine-*N*-oxide B (16)	Population	4	0.00366	0.07	0.9901
	Error Population × Replicates	10	0.05338		
	Time	2	1.04559	27.09	0.0000
	Population × Time	8	0.05152	1.33	0.2859
	Error Population × Replicates × Time	19	0.03860		
3′-*O*-Acetylechiumine-*N*-oxide (17)	Population	4	0.1233	0.93	0.4826
	Error Population × Replicates	10	0.1320		
	Time	2	19.4251	213.78	0.0000
	Population × Time	8	0.0863	0.95	0.5013
	Error Population × Replicates × Time	19	0.0909		

Table S4. Results of repeated measures ANOVA performed in Statistix 9 software for the data collected in controlled conditions experiment with *Echium plantaginuem* and *E. vulgare* harvested at 2 time points. Numbers 1-17 denote pyrrolizidine alkaloids and their N-oxides profiled in the study (Table 1).

Compound	Source GH2	df	Means Square	*F* Value	*p* Value
Leptanthine-*N*-oxide (1)	Species	1	90.1425	30.40	0.0003
	Error species × Replicates	10	2.9654	-	-
	Time	1	9.9146	3.22	0.1032
	Species × Time	1	0.9350	0.30	0.5939
	Error Species × Replicates × Time	10	3.0833	-	-
Echimiplatine-*N*-oxide (2)	Species	1	2.11244	28.72	0.0003
	Error species × Replicates	10	0.07356	-	-
	Time	1	0.45123	3.47	0.0922
	Species × Time	1	0.41397	3.18	0.1048
	Error Species × Replicates × Time	10	0.13010	-	-

Table S4. *Cont.*

Compound	Source GH2	df	Means Square	*F* Value	*p* Value
Uplandicine-*N*-oxide (3)	Species	1	3.90412	27.38	0.0004
	Error species × Replicates	10	0.14259	-	-
	Time	1	0.77926	6.57	0.0282
	Species × Time	1	1.08452	9.14	0.0128
	Error Species × Replicates × Time	10	0.11863	-	-
Intermedine-*N*-oxide (4)	Species	1	93.7158	119.92	0.0000
	Error species × Replicates	10	0.7815	-	-
	Time	1	1.5267	0.49	0.4991
	Species × Time	1	14.2565	4.59	0.0577
	Error Species × Replicates × Time	10	3.1043	-	-
Lycopsamine-*N*-oxide (5)	Species	1	132.312	146.02	0.0000
	Error species × Replicates	10	0.906	-	-
	Time	1	26.047	28.44	0.0003
	Species × Time	1	17.648	19.27	0.0014
	Error Species× Replicates × Time	10	0.916	-	-
7-Angeloylretronencine-*N*-oxide (6)	Species	1	8.67097	29.48	0.0003
	Error species × Replicates	10	0.29413	-	-
	Time	1	0.41990	3.07	0.1105
	Species × Time	1	2.00264	14.62	0.0034
	Error Species × Replicates × Time	10	0.13699	-	-

Table S4. *Cont.*

Compound	Source GH2	df	Means Square	*F* Value	*p* Value
	Species	1	111.730	160.57	0.0000
	Error species × Replicates	10	0.696	-	-
7-*O*-Acetyllycopsamine\intermedine (7)	Time	1	5.271	7.36	0.0218
	Species × Time	1	5.271	7.36	0.0218
	Error Species × Replicates × Time	10	0.716	-	-
	Species	1	61.6323	16.39	0.0023
	Error species × Replicates	10	3.7612	-	-
7-*O*-Acetyllycopsamine\intermedine-*N*-oxide A (8)	Time	1	2.5286	0.70	0.4238
	Species × Time	1	10.7843	2.97	0.1158
	Error Species × Replicates × Time	10	3.6360	-	-
	Species	1	107.996	240.20	0.0000
	Error species × Replicates	10	0.450	-	-
7-*O*-Acetyllycopsamine\intermedine-*N*-oxide B (9)	Time	1	13.843	4.51	0.0597
	Species × Time	1	7.472	2.43	0.1499
	Error Species × Replicates × Time	10	30.712	3.071	-
	Species	1	4.68001	15.23	0.0030
	Error species × Replicates	10	0.30737	-	-
9-*O*-Angelylretronencine-*N*-oxide (10)	Time	1	0.05202	0.22	0.6512
	Species × Time	1	2.20919	9.22	0.0125
	Error Species × Replicates × Time	10	0.23954	-	-
	Species	1	1.98223	24.94	0.0005
	Error species × Replicates	10	0.07948	-	-
Echimidine-*N*-oxide A (11)	Time	1	0.17674	1.06	0.3272
	Species × Time	1	0.90413	5.43	0.0420
	Error Species × Replicates × Time	10	0.16653	-	-

Table S4. *Cont.*

Compound	Source GH2	df	Means Square	*F* Value	*p* Value
Echiuplatine-*N*-oxide (12)	Species	1	0.00002	0.00	0.9974
	Error species × Replicates	10	1.96388	-	-
	Time	1	0.00002	0.00	0.9974
	Species × Time	1	3.92773	2.00	0.1877
	Error Species × Replicates × Time	10	1.96388	-	-
Echimidine-*N*-oxide B (13)	Species	1	0.03547	0.01	0.9068
	Error species × Replicates	10	2.45940	-	-
	Time	1	4.66203	1.96	0.1918
	Species × Time	1	0.39405	0.17	0.6926
	Error Species × Replicates × Time	10	2.37922	-	-
3′-*O*-Acetylechimidine-*N*-oxide (14)	Species	1	61.2240	16.42	0.0023
	Error species × Replicates	10	3.7286	-	-
	Time	1	12.0191	5.92	0.0352
	Species × Time	1	11.2381	5.54	0.0404
	Error Species × Replicates × Time	10	2.0287	-	-
Echiumine-*N*-oxide A (15)	Species	1	136.259	34.77	0.0002
	Error species × Replicates	10	3.919	-	-
	Time	1	0.286	0.11	0.7436
	Species × Time	1	10.638	4.21	0.0674
	Error Species × Replicates × Time	10	2.529	-	-
Echiumine-*N*-oxide B (16)	Species	1	27.8087	24.95	0.0005
	Error species × Replicates	10	1.1145	-	-
	Time	1	3.2958	2.68	0.1329
	Species × Time	1	0.0102	0.01	0.9292
	Error Species × Replicates × Time	10	1.2315	-	-
3′-*O*-Acetylechiumine-*N*-oxide (17)	Species	1	98.0422	119.87	0.0000
	Error species × Replicates	10	0.8179	-	-
	Time	1	15.4072	19.55	0.0013
	Species × Time	1	1.7363	2.20	0.168
	Error Species × Replicates × Time	10	0.7880	-	-

Table S5. Echium plantagineum samples collected from glasshouse experiment in the study of phenological cycles for each population including: Bendigo, Cobar, Coombah, Grenfell, Silverton, and Wagga Wagga.

Plant Age (Weeks)	Phenological State	Block of Replication
1	Seedling	1
1	Seedling	2
1	Seedling	3
7	Rosette	1
8	Rosette	2
9	Rosette	3
12	Flowering	1
13	Flowering	2
14	Flowering	3

Table S6. Plant samples obtained from controlled conditions experiment using two populations of *E. plantagineum* and two populations of *E. vulgare*, harvested at two time points. Each sample was a composite of four plant extracts. At both harvest dates, *E. vulgare* was at rosette stage.

Species	Location	Plant Age (Weeks)	Phenological State	Block
	Adelong 1	6	Rosette	1
E. plantagineum	Adelong 1	27	Flowering	1
	Adelong 2	7	Rosette	2
	Adelong 2	28	Flowering	2
	Adelong 3	8	Rosette	3
	Adelong 3	29	Flowering	3
	Silverton 1	6	Rosette	1
E. plantagineum	Silverton 1	27	Flowering	1
	Silverton 2	7	Rosette	2
	Silverton 2	28	Flowering	2
	Silverton 3	8	Rosette	3
	Silverton 3	29	Flowering	3

Table S6. *Cont.*

Species	Location	Plant Age (Weeks)	Phenological State	Block
E. vulgare	Adaminaby 1	6	Rosette	1
	Adaminaby 1	27	Rosette	1
	Adaminaby 2	7	Rosette	2
	Adaminaby 2	28	Rosette	2
	Adaminaby 3	8	Rosette	3
	Adaminaby 3	29	Rosette	3
	Cooma 1	6	Rosette	1
	Cooma 1	27	Rosette	1
	Cooma 2	7	Rosette	2
	Cooma 2	28	Rosette	2
	Cooma 3	8	Rosette	3
	Cooma 3	29	Rosette	3
